# Geometric Accuracy Assessment of AI-Based Auto-contouring Using OncoStudio for Prostate Cancer Treatment Planning

**DOI:** 10.7759/cureus.94067

**Published:** 2025-10-07

**Authors:** Masataka Hoshina, Masumi Kawaguchi, Shinji Sugahara, Masato Takanashi, Masaya Noguchi, Ayaka Oosato, Kouichi Masuda

**Affiliations:** 1 Department of Radiology and Radiation Oncology, Tokyo Medical University Ibaraki Medical Center, Ami, JPN; 2 Department of Nursing, Tokyo Medical University Ibaraki Medical Center, Ami, JPN

**Keywords:** ai, artificial intelligence, auto-contouring, auto-segmentation, prostate cancer, treatment planning

## Abstract

Artificial intelligence (AI)-based auto-contouring techniques in radiation treatment planning require thorough clinical validation. In this study, we evaluated the geometric similarity between AI-generated auto-contours and manually drawn contours in prostate cancer treatment plans for 15 patients using OncoStudio (Oncosoft, Seoul, South Korea). The mean Dice similarity coefficients for the bladder, rectum, and right and left femoral heads were 0.90, 0.95, 0.97, and 0.97, respectively. The mean 95th percentile Hausdorff distances were 7.3 mm, 7.1 mm, 5.6 mm, and 5.0 mm for the same structures. These results demonstrate that OncoStudio’s auto-segmentation provides high-quality contours suitable for prostate cancer treatment planning.

## Introduction

Intensity-modulated radiation therapy (IMRT) provides a conformal dose distribution, even in cases where the tumor is close to surrounding normal tissues or has a complex shape. By creating steep dose gradients at the boundaries between the tumor and nearby organs at risk (OARs), IMRT is expected to reduce side effects and improve treatment outcomes compared with conventional radiation therapy.

Accurate dose delivery requires precise identification of both the target and the OARs; therefore, accurate contouring is essential. Inaccurate contouring can lead to suboptimal clinical outcomes and increased side effects. When performing IMRT for head and neck cancer, numerous anatomically complex surrounding organs must receive sufficiently low doses. The contouring process can take from one to several hours, placing a heavy burden on treatment planners [1]. Manual delineation also results in variability between planners [2], and contouring accuracy directly influences treatment outcomes [3].

In recent years, research and development of artificial intelligence (AI) have expanded rapidly in the field of radiation therapy. AI offers the potential to automate and optimize the complex and time-consuming process of contouring and treatment planning. Auto-contouring of normal tissues can reduce workload and variability in the treatment process. Studies have reported that AI-based auto-contouring shortens contouring time and decreases interobserver variability [4-7]. We have been evaluating a commercial AI auto-contouring software, OncoStudio (Oncosoft, Seoul, Korea), for which few validation studies have been published.

This study aimed to assess the geometric accuracy of AI-based auto-contouring using OncoStudio.

## Materials and methods

CT data from 15 patients with high-risk localized prostate cancer were acquired between January 2023 and March 2025 using a SOMATOM Emotion 16 scanner (Siemens Healthineers, Forchheim, Germany) with a pelvic protocol (110 kVp, 300 mAs, 1-mm slice thickness, CareDose 4D). Eligibility criteria were as follows: (1) histologically confirmed prostate cancer; (2) completion of definitive volumetric-modulated arc therapy (VMAT) with a total dose of 76 Gy in 38 fractions; (3) availability of treatment planning CT images; and (4) diagnostic image quality sufficient for analysis. Exclusion criteria included re-irradiated cases and images containing artifacts due to prostheses. Patients were instructed to maintain a full bladder one hour before the CT scan.

Table 1 summarizes the demographics of the 15 patients included in this study. The median age was 81 years (range, 62-87 years), median height 167 cm (range, 154-173 cm), median weight 66 kg (range, 54.5-78 kg), and median body mass index (BMI) 24.8 (range, 19.5-28.7). To compare automated and manual segmentation, radiation oncologists manually contoured the organs at risk (OARs) surrounding the prostate, namely, the bladder, rectum, and femoral heads. AI-based auto-segmentation was performed using OncoStudio version 2.0. Manual contours were transferred from Pinnacle to OncoStudio for comparison. This study was approved by the Ethics Committee of Tokyo Medical University (approval number: T2025-0017).

**Table 1:** Demographics of 15 prostate cancer patients involved in this study

	Median	Range
Age (year)	81	62-87
Height (cm)	167	154-173
Weight (kg)	66	54.5-78
BMI	24.8	19.5-28.7

Using OncoStudio’s deep learning algorithm and patient CT images, automated contouring was performed for the OARs, including the bladder, rectum, and left (L) and right (R) femoral heads. These automatically generated contours were compared with those manually delineated by radiation oncologists with over 30 years of clinical experience. To evaluate the agreement between automated and manual segmentations, we used the Dice Similarity Coefficient (DSC) and the 95th percentile Hausdorff Distance (HD95). The DSC represents the degree of overlap between auto-contoured and manually contoured regions, ranging from 0 to 1, with values closer to 1 indicating greater similarity. The HD95 represents the 95th percentile distance (in millimeters) between the two contours.

## Results

Table 2 presents the DSC and HD95 values for the bladder, rectum, and L and R femurs among the 15 patients. For each organ, the DSC and HD95 were calculated per patient, and the minimum, maximum, mean, and standard deviation (SD) were summarized. The mean DSCs for the bladder, rectum, and R and L femurs were 0.90, 0.95, 0.97, and 0.97, respectively. These results indicate that the AI-based OAR contouring demonstrated a high level of agreement with manual segmentation.

**Table 2:** DSC and HD95 for the bladder, rectum, and left and right femurs among the 15 patients DSC: Dice Similarity Coefficient, HD95: 95th percentile Hausdorff Distance, L: left, R: right.

	DSC				HD95 (mm)			
Patient	Bladder	Rectum	Femur_L	Femur_R	Bladder	Rectum	Femur_L	Femur_R
1	0.817	0.954	0.967	0.970	10.323	5.368	5.612	3.937
2	0.913	0.933	0.968	0.969	5.813	16.073	5.816	4.136
3	0.920	0.968	0.968	0.967	5.550	4.030	4.388	8.004
4	0.945	0.942	0.970	0.974	5.752	5.284	7.935	3.246
5	0.939	0.949	0.970	0.971	3.410	5.301	3.719	3.410
6	0.918	0.963	0.959	0.967	4.358	6.079	5.000	4.984
7	0.920	0.956	0.967	0.967	7.465	6.345	4.049	3.096
8	0.850	0.941	0.964	0.961	9.630	11.590	4.606	10.303
9	0.877	0.947	0.972	0.973	10.742	9.132	4.496	3.010
10	0.901	0.972	0.963	0.966	5.814	4.232	6.191	6.866
11	0.925	0.943	0.970	0.956	6.607	8.004	4.626	8.890
12	0.900	0.960	0.961	0.965	13.002	3.751	5.995	4.049
13	0.887	0.924	0.970	0.970	9.129	12.002	4.803	7.813
14	0.950	0.966	0.970	0.969	3.410	3.580	4.262	6.601
15	0.879	0.960	0.968	0.965	7.813	5.261	3.134	5.412
Min	0.817	0.924	0.959	0.956	3.410	3.580	3.134	3.010
Max	0.950	0.972	0.972	0.974	13.002	16.073	7.935	10.303
Mean	0.903	0.952	0.967	0.967	7.255	7.069	4.976	5.584
SD	0.036	0.014	0.004	0.005	2.826	3.639	1.183	2.349

The mean HD95 values were 7.3 mm, 7.1 mm, 5.6 mm, and 5.0 mm for the bladder, rectum, and R and L femurs, respectively. The maximum deviations were slightly larger for the bladder and rectum but remained within a clinically acceptable range. In the tables and figures presented in this article, “femurs” refer to the femoral heads for brevity.

**Figure 1:**
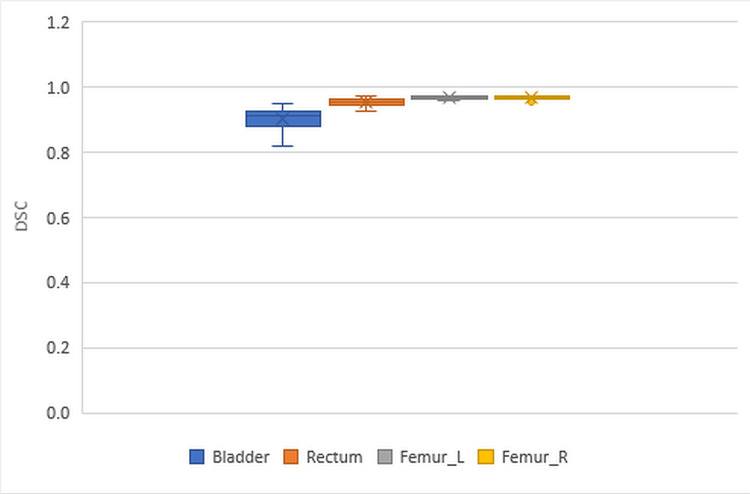
Box plots of DSC for the bladder, rectum, and left and right femurs DSC: Dice Similarity Coefficient, L: left, R: right.

Figure 1 shows the box plots of the DSC values for the bladder, rectum, and L and R femurs. Each colored box represents the 25th to 75th percentiles and the median, while the error bars indicate the minimum and maximum values. The DSC values range from 0 to 1.　

**Figure 2:**
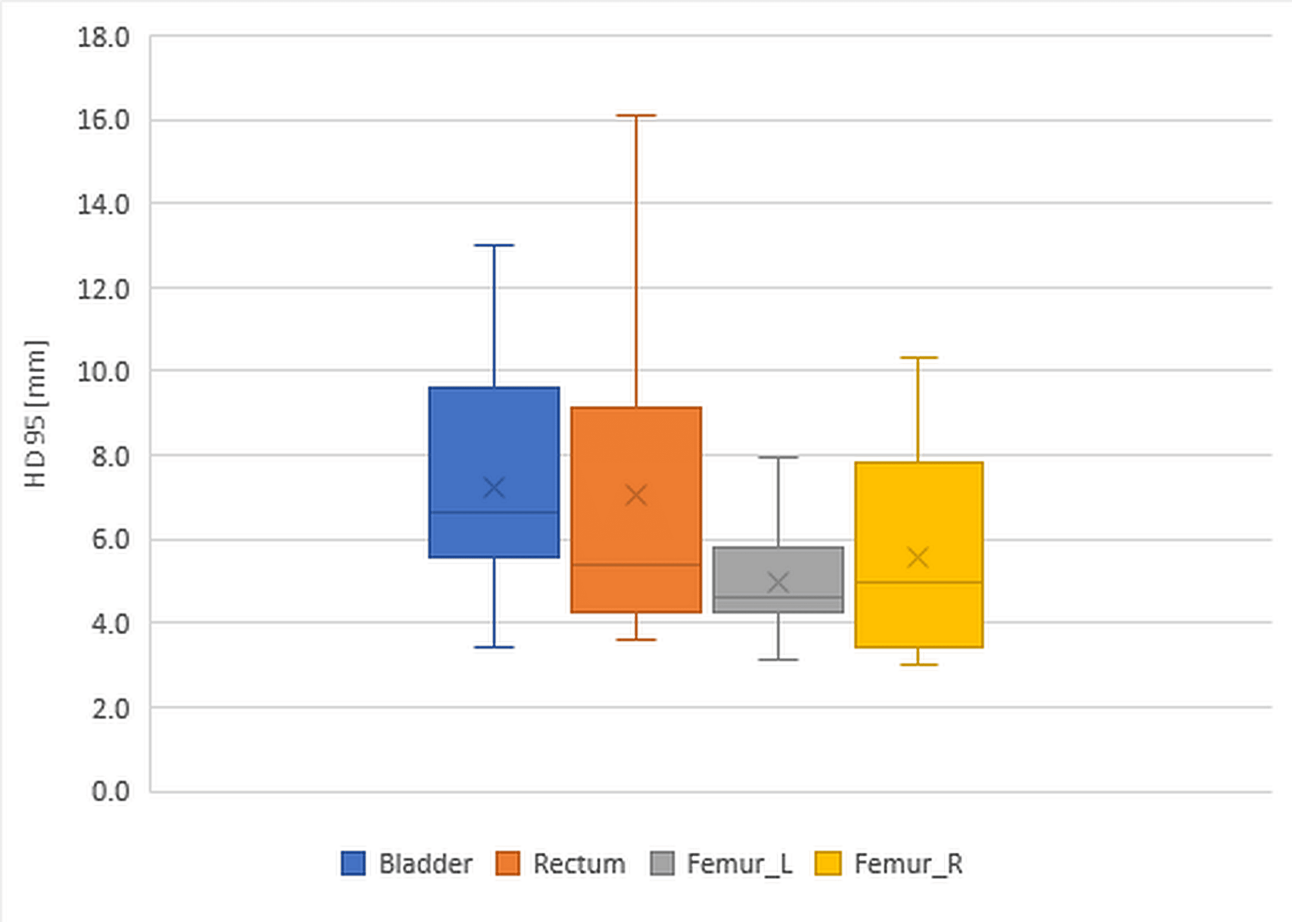
Box plots of HD95 for the bladder, rectum, and left and right femurs HD95: 95th percentile Hausdorff Distance, L: left, R: right.

Figure 2 presents the box plots of 95th percentile Hausdorff Distances (HD95) for the bladder, rectum, and L and R femurs. Each box represents the 25th to 75th percentiles with the median line, and the error bars indicate the minimum and maximum values. HD95 is measured in millimeters (mm).

**Figure 3:**
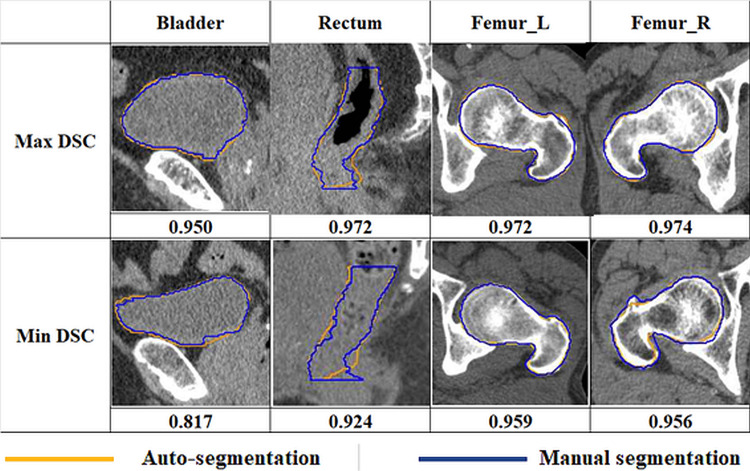
Comparison of the auto-contoured and manually contoured data for the bladder, rectum, and left and right femurs DSC: Dice Similarity Coefficient, L: left, R: right.

Figure 3 shows the comparison of the auto-contoured and manually contoured data for the bladder, rectum, and L and R femurs, showing the maximum and minimum DSC values among the 15 patients. Overlaid yellow and blue contours represent the auto-segmentation and manual segmentation results, respectively.

## Discussion

In this study, we used OncoStudio to evaluate the DSC between AI-based auto-contouring and manual contouring in 15 patients with high-risk localized prostate cancer. Previous studies have reported DSC values ranging from 0.85 to 0.96 for the bladder and 0.72 to 0.96 for the rectum, indicating that AI-based contouring can substantially reduce interobserver variability [8,9]. In the present study, the mean DSCs ranged from 0.90 to 0.97, consistent with these earlier findings [8,9]. Notably, our results showed higher DSCs for the rectum.

The standard deviation (SD) of DSC values across the 15 patients was small for all OARs (the bladder, rectum, and both femoral heads), ranging from 0.004 to 0.036. These small SDs indicate the stability and consistency of the automated contouring. The lowest SDs were observed in both femoral heads, likely because these structures have high contrast with adjacent tissues and similar shapes among patients.

DSC comparison metrics are also useful for identifying cases with low similarity to the final approved contours. Previous studies have reported that bladder contours may be inaccurate when adjacent bowel or prostate tissue is included, and rectal contouring can be affected by large amounts of intraluminal gas [9-11]. Similar findings were observed in this study, which may explain why the SDs of DSC values for the bladder and rectum were slightly higher than those for the femoral heads.　

In our study, the DSC for the bladder ranged from 0.82 to 0.95. In some cases, the bladder showed relatively lower DSC values compared to other OARs, possibly due to slight contour discrepancies at the borders with the small bowel and the protruding portion of the prostate.

Previous studies have reported that AI-based rectum segmentation generally yields lower DSC values than other organs, and that rectal contours often require more extensive manual editing compared to the bladder [12]. The lower DSC values in automatic rectum contouring are attributed to the rectum’s complex anatomical characteristics, including shape variability, positional changes, and poor image contrast [13].

In this study, the DSC for the rectum ranged from 0.92 to 0.97, demonstrating high values with small standard deviations. A detailed review of the case with the lowest DSC among the 15 patients revealed discrepancies in organ recognition at the border with the sigmoid colon and deviations from manual contours in low-contrast soft tissue regions such as the anal sphincter. The DSC values appeared to vary depending on whether a particular intestinal segment was identified as the rectum or the sigmoid colon. These findings suggest that the rectum’s complex anatomical characteristics may contribute to inaccuracies in automatic contouring. Manual correction in areas with low contrast, particularly at the borders with the sigmoid colon and anal sphincter, could potentially further improve DSC accuracy.

AI-based automatic contouring has been reported to reduce interobserver variability compared to manual contouring [8]. Its clinical acceptability is also high, with 95.7% of AI-generated contours rated as “perfect” or “acceptable,” suggesting equivalence or superiority to contours created by radiation oncologists [9]. In this study, the high mean DSC and low SD values for all OARs indicate strong agreement between automated and manual contours, demonstrating high reproducibility. AI-based automated contouring for prostate radiation therapy has shown significant clinical benefits in previous studies, including improved workflow efficiency and practical utility [8,14]. For the OARs examined in this study, sufficient similarity was achieved, suggesting that clinical use is feasible with only minimal manual adjustments in areas of reduced similarity, thereby substantially reducing workload.

Our study has several limitations. First, the sample size was small (N = 15), which may limit the reliability and generalizability of the statistical analysis. Second, this was a single-institution, retrospective study. Third, the small number of cases collected over two years may have introduced selection bias. These factors could affect the generalizability of our findings. Further studies with larger, multicenter, prospective designs are warranted to validate these results.

## Conclusions

Auto-segmentation provided high-quality contours for prostate cancer patients, demonstrating good agreement with manual segmentation. Automating CT-based organ contour extraction for prostate cancer treatment planning proved feasible and performed well in clinical settings. These findings highlight its potential as a reliable and efficient tool to support prostate radiotherapy planning. Automated contouring, followed by manual correction, offers multiple advantages, including reduced inter-planner variability and improved workflow efficiency. Because contouring accuracy directly affects plan optimization, evaluation, and clinical decision-making, automated contouring alone should not be used without expert review. A qualified physician must always evaluate and, if necessary, adjust the contours to ensure precision. In clinical practice, accurate AI-based automated contouring can streamline treatment planning by standardizing contouring criteria while reducing workload. Our results suggest that integrating automated contouring into the prostate treatment planning process is clinically feasible and may contribute to establishing a standardized and efficient clinical workflow.
